# Headache and migraine in mitochondrial disease and its impact on life—results from a cross-sectional, questionnaire-based study

**DOI:** 10.1007/s13760-021-01630-4

**Published:** 2021-03-08

**Authors:** Philipp Burow, Anneke Meyer, Steffen Naegel, Stefan Watzke, Stephan Zierz, Torsten Kraya

**Affiliations:** 1 Department of Neurology, University Hospital Halle-Saale, Ernst-Grube-Str. 40, 06120 Halle (Saale), Germany; 2 Clinic for Psychiatry, Psychotherapy and Psychosomatics, University Hospital Halle-Saale, Julius-Kühn-Str.7, 06112 Halle (Saale), Germany; 3 Department of Neurology, Hospital Sankt Georg Leipzig gGmbH, Delitzscher Str. 141, 04129 Leipzig, Germany

**Keywords:** Headache, Migraine, Mitochondrial disease, CPEO

## Abstract

Headache is frequent in patients with mitochondrial disorders. Previous studies point to a higher prevalence of headache in these patients than in the general population. As mitochondrial disorders often present a variety of other symptoms, the question arises how much the presence of headache really influences daily life. We performed a cross-sectional, questionnaire-based study investigation with 61 patients with a genetically proved mitochondrial disease mainly composed of CPEO phenotype. Headache was examined using a standardized questionnaire, and classified according to ICHD-2. Headache-related disability was evaluated by the Headache-Impact-Test-6 (HIT-6). Additionally, depression and anxiety were examined using the Hospital Anxiety and Depression Scale (HADS) and Short-Form-Health Survey (SF-12) was used to investigate the health-related quality of life. Headache was reported by 43/61 (70.5%) of the patients. 35/61 patients (57.4%) described a Tension-type headache (TTH) and 26 patients (42.6%) a migraine. Patients reporting headache had a significantly higher HIT-6 score than those without (mean: 54.47 vs. 38.47, *p* < 0.001). The HIT-6 score was significantly higher in patients reporting a migraine compared to those with a tension-type headache (mean: 62.13 vs. 46.18, *p* < 0.001). In the HADS score and in the SF-12 were not significantly influenced by the occurrence of headache. This study confirms the previously reported frequent occurrence of headache in a large cohort of patients with a confirmed mitochondrial disease. Migraine had the greatest impact on daily living, which appeared not to be confounded by depression and anxiety. Thus, we conclude that Migraine may be a substantial contributor for burden of disease in patients with mitochondrial diseases.

## Introduction

Mitochondrial diseases (MDs) are multisystemic disorders frequently affecting the brain. The spectrum of symptoms is broad and include chronic progressive external ophthalmoplegia (CPEO), the syndrome of mitochondrial encephalomyopathy, lactic acidosis and stroke-like episodes (MELAS), and myoclonic epilepsy and ragged red fibers syndrome (MERRF). Genetically, point mutations of the mitochondrial DNA (mtDNA, eg. M3243 G > A and m.8344 A > G), large-scale single mtDNA deletions and mutations in the nuclear DNA (eg. POLG) are frequently found [1]. There is a high variability of genotypes and phenotypes in mitochondrial diseases generating a challenging diagnostic and therapeutic management [2].

Headache is a common symptom in patients with MDs [3] and it is still under debate whether this reflects coincident primary headache disorders, a secondary migraine- or tension-type-like headache caused by a mitochondrial disease or a mix of both. For simplification, we use the terms migraine and tension-type headache throughout the whole manuscript. In many cases, the criteria of a migraine according to the International Classification of Headache Disorders (ICHD-3) are fulfilled [4]. Only for the MELAS-syndrome, a secondary headache attributed to a genetic vasculopathy is established [5]. However, in a recent study, we detected a higher prevalence of headache than one would expect in the general population in a cohort of patients with a mitochondrial disease dominantly composed of the CPEO phenotype [3]. In this cohort, tension-type headache was more frequent than migraine. Up to now, the treatment is symptomatic and follows the recommendations for primary headache disorders. According to the International Classification of Functioning, Disability and Health (ICF), a multimodal approach including measurements of life quality and participation in daily life is helpful for an adequate treatment of patients with a complex disease [6]. Thus, we conducted this study, in which not only the prevalence of headache, but also the impact of headache on daily life, health-related quality of life and symptoms of depression and anxiety are investigated using a standardized questionnaire in a cohort of patients with a genetically proven mitochondrial disease.

## Methods

### Recruitment

Patients were recruited from the database of the Department of Neurology, University Hospital Halle (Saale) by the following inclusion criteria: (1) genetically proven mitochondrial disease and (2) patient is able to complete the headache questionnaire sufficiently. All patients had previously attended the special consultation for Mitochondrial Disorders at the Department of Neurology and had undergone all necessary clinical, laboratory and genetic testings. All analyzed patients gave written informed consent for the survey. The study protocol was approved by the local ethics committee (University Halle-Wittenberg). Data collection was conducted in the years 2014 and 2015 for a duration of 12 months.

### Questionnaire

All patients answered the “German headache questionnaire” that was previously published and validated and corresponds to the International Criteria of Headache Disorders (ICHD-2) to screen for primary headaches (migraine, cluster headache, and tension-type headache) [7]. Consistent with the concept of the used questionnaire, in patients describing more than one type of headache, all different headache diagnoses were classified. Patients were asked regarding the headache prevalence within the last 12 months, as well as the lifetime prevalence. Additionally, the use of pain killers for the treatment of acute headache attacks was investigated including the name of the product(s) and the number of days a month on which pain killers were taken.

## Statistical analysis

The sample sizes are based on a convenience sample, and, since numbers are counted, no a priori statistical power calculation was conducted. The analyses are descriptive in nature. The term prevalence was used to describe proportions in this survey. This should be noted for interpretation. Explorative statistical analyses were performed using SPSS 17.0 (International Business Machines Corporation, Armonk, New York, USA) for Microsoft Windows. The statistical tests used are indicated within the sections. The level of significance for hypothesis testing was set to *P* = 0.05.

## Results

### Study population

Questionnaires were sent to 130 patients with a genetically proved mitochondrial disease from the database of our tertiary neuromuscular centre. While 17 returned being undeliverable. Of the 113 remaining, 52 could not be included, as 39 sent were back without written consent, and 13 questionnaires were incompletely filled out and thus not evaluable. We were able to analyze questionnaires of 61 German patients (26 male, 35 female). The mean age of the cohort was 46.7 years (range 24–83 years). The following distribution of gene mutations was found: 28 patients (45%; 9 male, 19 female) with single mtDNA deletions, 20 patients (33%; 10 male, 10 female) with multiple mtDNA deletions, 11 patients (19%; 7 male, 5 female) with point mutations of the mtDNA (m.11778G > A, m.3243A > G, m.5591G > A, m.3460G > A, m.622G > A, ND1, ND5), and 2 patients with heterozygous nuclear mutations (POLG 1 and 2). Phenotypically, the cohort consisted of 40 patients with CPEO or CPEO plus (66%; 15 male, 25 female), 8 suffering mitochondrial myopathy (13%; 6 female, 2 male), 5 suffering LHON (8%; 4 female, 1 male), 2 patients with a diagnosed MELAS-syndrome (3%; 1 male, 1 female), one suffering SANDO-syndrome (2%) and 5 without distinct phenotype (8%).

### Frequency and distribution of headache

In the study population, 71% (43/61) indicated headache and 29% (18/61) reported not to suffer any headache. Evaluation of the questionnaires revealed a clear tension-type headache in 27% (17/61) of the patients. 43% (26/61) reported a migraine fulfilling all criteria according to ICHD-2. Of these, 18 patients (30% of the whole cohort) reported a combination of migraine and tension-type-headache and 8 patients (13%) a pure migraine without any other headache. 50% of all migraine patients reported aura symptoms (13/26).

In the CPEO subgroup, 71% (28/39) indicated headache with the following distribution: pure tension-type headache in 23% (9/39), migraine overall 49% (19/39), 36% (14/39) both migraine and tension-type headache. The prevalence of pure migraine was identically distributed as in the whole cohort with 13% (5/39). These results are summarized in Table 1.

**Table 1:** Frequency of headache subtypes of the whole study cohort in comparison to the CPEO subgroup

	Whole cohort (*n* = 61)		CPEO subcohort (*n* = 39)	
No headache	18/61 (29%)		11/39 (29%)	
Headache (any)	43/61 (71%)		28/39 (71%)	
Migraine and TTH	18/61 (29%)	26/61 (43%)	14/39 (36%)	19/39 (49%)
Pure migraine	8/61 (13%)		5/39 (13%)	
Pure TTH	17/61 (27%)		9/39 (23%)	

The percentage values were always calculated in relation to the corresponding cohort. The total number of migraine patients in each cohort is given on the right side. For pure migraine, migraine and tension-type headache (TTH) a summed value is given, as migraine and TTH may be chronic migraine (according to ICHD-3)

### Impact of headache on daily life

**Fig. 1:**
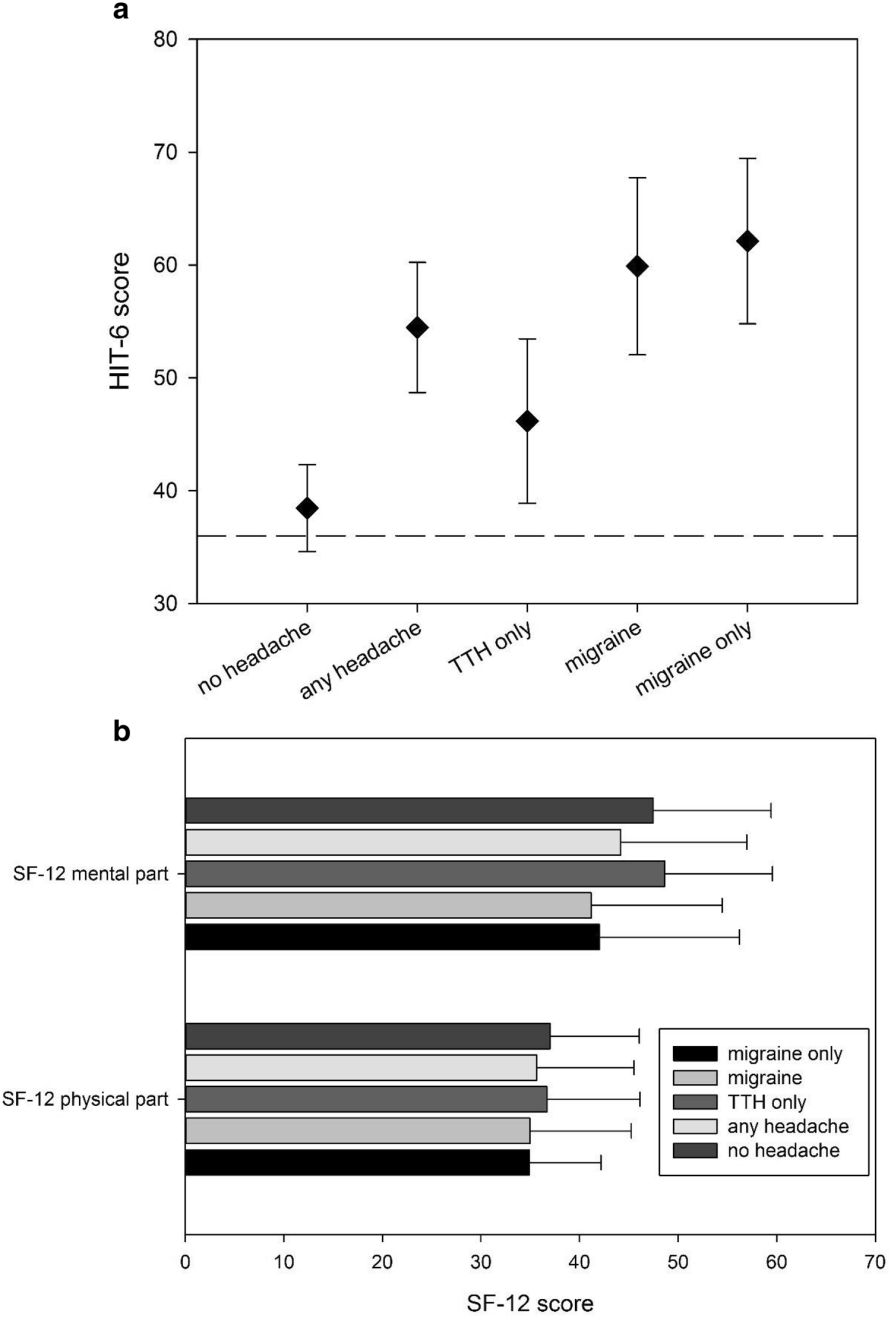
Results of the HIT-6 and SF-12 tests of the whole cohort depending on the headache diagnosis. All bars in the graph show mean values and standard deviations. For the number of patients, we refer to Table 1. The statistical analysis is presented in the text. **a** HIT-6 values of the indicated subgroups. The group “only migraine” includes all patients with a migraine without a tension-type headache as diagnosed from the questionnaire and is thus a subgroup of “migraine”. The dashed reference line displays the minimum score of the HIT-6 (36 points). **b** SF-12 values separately for both the mental and the physical part

For assessment of the impact of headache on daily life, the HIT-6 questionnaire was used. The results are shown in Fig. 1a. The HIT-6 score was significantly higher in patients with headache (54.47, 95%-CI: 47.91–61.12) compared to those without (38.47, 95%-CI: 36.49–40.45; two-sided significance test: *p* < 0.001). In the headache group, the highest score was reached in the subgroup with migraine without tension-type headache (62.13, 95%-CI: 55.99–68.26) followed by the subgroup reporting both (58.89, 95%-CI: 54.87–62.91). In the tension-type headache group without migraine, the values were lower (46.18, 95%-CI: 42.43–49.92). Due to the overlapping headache groups, we performed a multivariate statistical analysis to assess the influence of the individual headache entities on the HIT-6. Only migraine had a significant impact on the HIT-6 value (*p* < 0.001, One-way-ANOVA), for tension-type headache no significant effect was detected (*p* = 0.579, One-way-ANOVA). The genotype and phenotype of the mitochondrial disease did not significantly influence the score of the HIT-6 (genotype: *p* = 0.829, phenotype: *p* = 0.427, One-Way-ANOVA).

### Health-related quality of life, anxiety, and depression

The results of the different parts of SF-12 and HADS are shown in Fig. 1b and Table 2. The presence of headache did not have any impact on both the physical and mental part of the SF-12 (physical part: *p* = 0.622, mental part: *p* = 0.359, One-way-ANOVA). In the subgroup analysis, patients with migraine trended toward a higher score in the mental part of SF-12 in comparison to those reporting only tension-type headache (physical part: *p* = 0.506, mental part: *p* = 0.063, One-way-ANOVA), but this failed to show significance. The analysis of the HADS scores did not reveal a difference between patients with headache and without (anxiety sum score: *p* = 0.576, depression sum score: *p* = 0.716, One-way-ANOVA). In patients reporting headache, the presence of migraine did not influence both parts of the HADS (anxiety sum score: *p* = 0.117, depression sum score: *p* = 0.258, One-way-ANOVA) in comparison to those only reporting a tension-type-headache (anxiety sum score: *p* = 0.831, depression sum score: *p* = 0.599, One-way-ANOVA).

**Table 2:** Results of the SF-12 and HADS questionnaire

	SF-12 (mental part)	SF-12 (physical part)	HADS: depression sum score	HADS: anxiety sum score
No headache (*n* = 18)	47.46 ± 11.98	36.99 ± 9.07	7.22 ± 5.07	6.17 ± 5.25
Any headache (*n* = 42)	44.19 ± 12.79	35.65 ± 9.88	7.74 ± 5.09	6.98 ± 5.08
Migraine and TTH (*n* = 25)	41.17 ± 13.27	34.95 ± 10.29	8.46 ± 5.38	7.96 ± 5.53
Pure migraine (*n* = 8)	42.00 ± 14.22	34.90 ± 7.27	6.88 ± 4.16	6.63 ± 4.17
Pure TTH (*n* = 17)	48.63 ± 10.95	36.68 ± 9.46	6.65 ± 4.57	5.47 ± 4.00

The values are given as mean ± standard deviation. The statistical analysis is given in the text

## Discussion

The main emphasis of this study was to analyze the headache prevalence, the health-related quality of life, the impact of headache in daily life and the occurrence of anxiety and depression with regard to headache in a cohort of patients with a confirmed mitochondrial disease. The study cohort (phenotype and genotype) was consistent with several other studies analyzing headache in patients with mitochondrial diseases. This study re-confirms the results of previous studies showing a high prevalence of headache in patients with a confirmed mitochondrial disease [3, 8].

In many patients, a combination of both diagnoses (migraine and tension-type headache) was made. From the data of the questionnaire, it is not possible to clearly distinguish between these two headache forms especially when both forms are present. Patients with severe migraine may consider migraine attacks with low headache intensity as tension-type headache although the criteria for migraine are fulfilled for most or all headache days. This is considered in the current concept of Chronic Migraine, which was introduced after ICHD-2 [5, 9, 10]. Thus, we additionally included all patients with both migraine and tension-type headache in the migraine group. We further were not able to analyze the headache days regarding the different headache forms for the same reason.

As a main finding, the HIT-6 score was significantly elevated in the subgroup of patients reporting a migraine, whereas this was not the case for TTH. This was independent of the genotype and phenotype of the mitochondrial disease. In patients with a mitochondrial disease, daily living appears to be influenced by the presence of migraine, but not by a tension-type-headache. As both parts of the HADS score did not show differences between the headache and non-headache group, we do not assume that the differences seen in the HIT-6 score were driven by depression or anxiety. These are frequent comorbidities in patients with mitochondrial diseases [11] and headache disorders [12, 13]. As we did not perform a face-to-face interview, we cannot completely rule out depression and anxiety disorders in our patients. With the results of the HADS score, we conclude that symptoms of depression and anxiety are not associated with the impact of migraine on daily life in our cohort.

The mental health may be influenced by the presence of migraine as indicated by a tendency in SF-12 in our cohort. This result must be handled with caution, as it was not statistically significant. It may be explained by the higher intensity of headache attacks and the presence of accompanying symptoms in patients with migraine in comparison to tension-type headache. Nevertheless this needs further evaluation in a larger sample. The scores in the physical part of the SF-12 did not differ between the headache and the non-headache group.

Our study cohort mainly consisted of patients with CPEO, followed by mitochondrial myopathy, LHON and MELAS-syndrome. Other studies focused on the prevalence of migraine, in contrast, we distinguished tension-type headache and migraine and analyzed these separately. Approximately, 70% of our cohort reported headache. Of these, 81% reported typical symptoms of a tension-type-headache and 60% typical symptoms of a migraine. A significant amount of 40% reported both headache types. Especially in regard to migraine, the prevalence is far higher than in the general population [14]. This also accounts for the CPEO subgroup with numerically even a higher rate of nearly 50%. Previous studies reported slightly lower rates but these were still higher than in the general population [15, 16]. Thus, migraine can be regarded as a frequent headache subtype of CPEO patients with single and multiple deletions. Up to now, only one study analyzing the burden of headache in patients with a mitochondrial disease was published [17]. In the cohort of Tiehuis and colleagues 55% of the Patients reported headache with 48% of the whole cohort fulfilling the ICHD-3 criteria for migraine. These patients indicated a lower headache-related quality of life. As a major difference between the cohorts, in our analysis, only one single patient showed the m.3243A > G mutation, which is frequent in MELAS. Both studies were also different regarding the phenotypes of mitochondrial disease. Furthermore we compared different headache subtypes. Despite the differences, the results of both studies regarding the burden of headache and the almost similar prevalence of migraine in both cohorts point toward a more generalizable statement regarding the burden of headache in mitochondrial disorders.

Up to now, the international headache classification (ICHD-3) only considers Migraine-like attacks as a part of an underlying mitochondrial disease for the MELAS-syndrome. A mitochondrial dysfunction may play a role in migraine pathophysiology. We currently are not able to distinguish between a primary headache and a symptomatic headache possibly caused by a mitochondrial disease in our cohort. Furthermore, symptoms of mitochondrial diseases (in our cohort, especially visual loss or impairment, myalgia, muscular weakness, epilepsy) can also influence life quality. But we were able to show that especially migraine (or migraine-like) headache can noticeably affect daily life of patients with mitochondrial disease, especially with the CPEO phenotype. It appears reasonable that the measured impact is caused by the headache and not by other symptoms of mitochondrial diseases. Furthermore, anxiety and depression do not seem to play a major role in this connection.

We conclude that especially migraine can be a substantial factor of disease severity in patients with a mitochondrial disease without significant influence of depression and anxiety as confounding factors. This emphasizes that headaches should be investigated in a standardized way in each patient with a suspected or confirmed mitochondrial disease, as adequate treatment could help to significantly improve the quality of life. The identification and standardization of treatment for headache in patients with mitochondrial disorders needs further clarification and may be an important subject of further investigations using a prospective study protocol. A recent published cross-sectional study provided initial data for this question [17]. Up to now, the treatment follows the guidelines for the primary headaches.

This study has some limitations: headache was analyzed using validated questionnaires. Therefore, we were able to identify the headache type, but not other parameters such as the number of headache days per month, the intensity of headache attacks, and the duration of attacks. Furthermore, a valid diagnosis of a migraine aura is not possible using questionnaires. Thus we did not further evaluate this parameter. The cross-sectional design of the study does not allow to detect changes in the course of the disease, which could be of interest, as symptoms in mitochondrial diseases are known to be fluctuating. Further research is needed to address if these fluctuations go hand-in-hand with the burden of headache in this dedicated patient cohort. Furthermore, it is unclear whether an analysis should focus on genotypes or phenotypes in MDs. As there is no strong genotype–phenotype correlation in MDs, we eventually decided to focus on phenotypes.

## Data Availability

The datasets generated and analyzed during the current study are available from the corresponding author on reasonable request.
